# Recent downhill course of COVID-19 at Rohingya refugee camps in Bangladesh: Urgent action solicited

**DOI:** 10.7189/jogh.11.03097

**Published:** 2021-09-04

**Authors:** Sakirul Khan, Sheikh Mohammad Fazle Akbar, Kazunori Kimitsuki, Nobuo Saito, Takaaki Yahiro, Mamun Al Mahtab, Akira Nishizono

**Affiliations:** 1 Department of Microbiology, Faculty of Medicine, Oita University, Yufu, Oita, Japan; 2 Department of Gastroenterology and Metabology, Ehime University Graduate School of Medicine, Toon, Ehime, Japan; 3 Department of Hepatology, Bangabandhu Sheikh Mujib Medical University, Dhaka, Bangladesh

The coronavirus disease 2019 (COVID-19) pandemic has disrupted the health care delivery systems worldwide. Currently, Asian countries have had an upsurge in infections due to the spread of the Delta variants of severe acute respiratory syndrome coronavirus 2 (SARS-CoV-2) that started in India in April 2021 [1]. Bangladesh, a South Asian country bordering India, has also been experiencing a surge of Delta variant outbreaks. According to the report by the Institute of Epidemiology, Disease Control and Research of Bangladesh, the Delta variant was detected in about 80% of samples in June 2021 [2]. Hospitals are experiencing a sharp rise in the number of critical patients with increasing fatalities [3]. As Bangladesh harbors over a million forcibly displaced Myanmar’s ethnic Rohingya population, the United Nations High Commissioner for Refugees (UNHCR) has cautioned about severe outbreaks of COVID-19 in the Rohingya refugee camps [4].

## ROHINGYA REFUGEES LIVE IN AN IDEAL ENVIRONMENT FOR SARS-COV-2 TRANSMISSION

Currently, over 1.3 million Rohingya refugees are living in highly congested camps with high risk of COVID-19 in Bangladesh [5]. The majority of them live in 34 extremely congested camps with poor access to water and sanitation, and very limited health services (Photo 1). Due to malnutrition and low immunization coverage, continuous outbreaks of various infectious diseases like measles, hepatitis C, human immunodeficiency virus, and diphtheria are prevailing in the Rohingya refugee camps [6-11]. A significant number of deaths raged through the Rohingya refugee camps due to ongoing outbreaks of infectious diseases [7-9]. Also, a high proportion of Rohingya refugees are suffering from noncommunicable diseases [12]. All these prevailing situations are challenging the capacity of the health care system to tackle the severe COVID-19 outbreaks in the Rohingya refugee camps.

**Figure:**
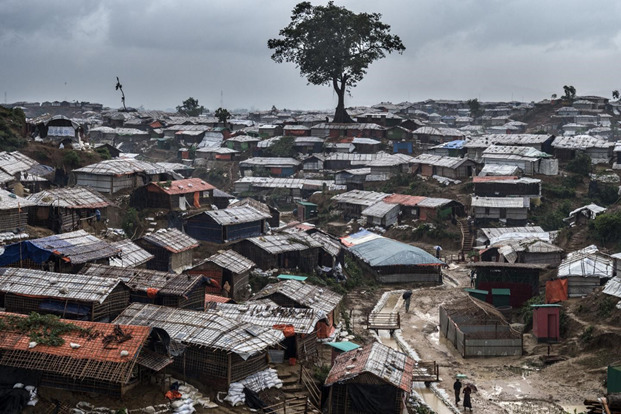
Photo: Rohingya pollution are living in the single largest and most densely populated cluster of refugee camps. Photo from UNICEF, Bangladesh (UNICEF/UN0235247/LeMoyne; Source: https://www.unicef.org/bangladesh/en/stories/three-years-rohingya-refugee-crisis).

The most effective responses to COVID-19 such as social distancing and quarantine concept are impossible in Rohingya refugee camps. The testing capacity is highly limited and treatment is usually late and inadequate. It has been estimated that a large-scale outbreak is very likely in the Rohingya camps with 70%–98% of the refugees expected to be infected, if no effective interventions are taken [13]. The numbers of COVID-19 cases and deaths increased considerably in the Rohingya refugee camps in June 2021 [14,15]. Any misinformation about COVID-19 may make the situation worse in the Rohingya refugee camps. Without appropriate information, equal and equitable health service provision as well as proper resource allocation is not possible. In this viewpoint, we highlighted the current COVID-19 situation in the Rohingya refugee camps and suggested means and ways for their protection. This will help the policymakers and international stakeholders to prioritize their efforts for these vulnerable populations.

## PRESENT STATUS OF COVID-19 IN ROHINGYA REFUGEE CAMPS

The first outbreak of COVID-19 in the Rohingya population was confirmed on May 14, 2020. A sharp increasing trend in cases among Rohingya refugees has been observed in July 2021. As of July 14th 2021, a total of 2147 patients from Rohingya refugees had COVID-19 and more than 70% of them were infected within the past few weeks [14]. Seven out of 34 camps (camp No. 1W, 2W, 3, 4, 15, 17, 24) reported more than 100 cases by early-July 2021. The median age of the COVID-19 patients was 22 (range 0–107) years and about 50% were men (Table 1). More than 80% of patients were under 40 years of old. Analyzing the severity of disease at presentation, about 15% of patients had moderate to severe disease with fever, cough, headache, and sore throat. However, the disease condition of 80% of patients was unknown (data missing or under reported).

**Table 1:** Characteristics of COVID-19 cases and deaths among Rohingya refugees*

Characteristics of cases	Numerical data	
Total number of cases	2147	
Number of cases over time:		
May 2020 – October 2020	336	
November 2020 – April 2021	242	
May 2021 – June 2021	1569	
Sex:		
Male (%)	1060 (49.4)	
Female (%)	1087 (50.6)	
Age, median (range) – years	22 (0-107)	
Age group (%):		
0-20	46	
21-40	37	
41-60	13	
>60	4	
Severity of disease at presentation:		
Critical (%)	2 (0.1)	
Severe (%)	26 (1.4)	
Mild/Moderate (%)	271 (12.6)	
Asymptomatic (%)	104 (4.8)	
Unknown (%)	1744 (81.2)	
**Characteristics of death:**		
Total number of deaths	20	
Number of deaths over time:		
May 2020 – October 2020	10	
November 2020 – April 2021	1	
May 2021 – June 2021	9	
Sex:		
Male (%)	12 (60)	
Female (%)	8 (40)	
Age group (%):		
0-20	5	
21-40	15	
41-60	45	
>60	35

*Data are from WHO reports (as of 14th July 2021 [14,15]).

**Figure 1:**
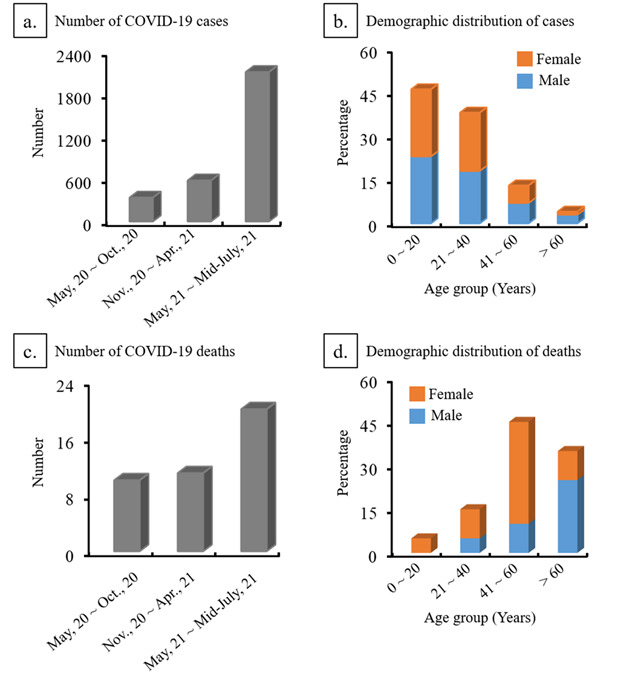
Number of cases and deaths for COVID-19 in Rohingya refugee camps. **Upper panel (a, b)**: Cumulative number of cases reported for SARS-CoV-2 over the time and their demographic distributions. **Lower panel (c, d)**: Cumulative number of deaths reported for COVID-19 over the time and their demographic distributions. Data are from WHO reports as of 14 July 2021 [14,15].

There have been 20 deaths in the Rohingya Refugees from COVID-19 and 60% were female (**Figure 1**). Fifty five percent of patients that died presented with severe conditions at the time of admission. In addition, 20% of deceased patients were under 30 years of age. In addition to COVID-19 confirmed deaths, more than 100 suspected acute respiratory infection-related deaths have been reported since the first outbreak occurred in the Rohingya refugee camps [15].

Currently, 572 general isolation beds are functional in isolation and treatment centers to assist both Rohingya refugees as well as the nearby host communities and over 60% of beds were occupied as of early July 2021. During the past few weeks, a sharp increase in bed occupancy was reported, indicating an increased demand for hospitalization due to severe disease presentation at admission. However, there are no Intensive Care Unit/High Dependency Unit (ICU/HDU) facilities available at refugee camps for severe and critical patients. Moreover, as of July 14th 2021, not a single dose of the COVID-19 vaccine has been administered to Rohingya refugees [4].

An ideal environment exists for the SARS-CoV-2 to spread in the highly densely populated (120 000 refugees per square miles) Rohingya refugee camps. The poor living conditions that make it impossible to maintain social distancing and quarantining, delayed diagnosis, reduced access to medicine, and absolute lack of advanced treatment facilities can lead to increased occurrence, severity and case fatality of COVID-19 in Rohingya refugee camps. Moreover, the deadly Delta variant of COVID-19 is spreading at an alarming level in the host communities surrounding refugee camps exacerbating the fears of massive outbreaks. In fact, the numbers of severe COVID-19 cases and deaths in Rohingya camps jumped three times in recent weeks. The pattern of present outbreaks indicates that the scenario in the Rohingya camps may be worsened in coming days. Modeling studies have predicted that without appropriate actions COVID-19 will spread unchecked within the Rohingya refugee camps, causing high levels of morbidity and mortality [13]. Therefore, to tackle the precarious situation and new infections in Rohingya refugees, there is an urgent need to increase capacity to provide adequate treatment and ensure the immediate rollout of vaccines against COVID-19.

## MORAL RESPONSIBILITY TO PROTECT THE MOST DEVASTATED AND NEGLECTED ROHINGYA REFUGEES

Mathematical modeling has reported that proper distribution of COVID-19 vaccines could reduce coronavirus deaths [16]. Vaccination against COVID-19 might be the most applicable measure in the Rohingya refugee population as the other effective responses such as testing, social distancing and quarantine are nearly impossible to implement in the camps. However, heterogeneous availability of COVID-19 vaccine among different countries or even the different groups of people in the same country have been reported [17,18]. For example, compared with high-income countries, the low-income countries that are hosting over 80% of refugees received only 0.3% of doses of COVID-19 vaccine [18,19]. However, they are unlikely to be distributed in refugee camps. In fact, less than 50, 000 of 82.4 million forcibly displaced people in 94 countries have been vaccinated against COVID-19 worldwide by June 2021 [20]. Most importantly, not a single Rohingya has received any vaccination against SARS-CoCV-2 till now. [4].

Bangladesh has begun the COVID-19 vaccination program nationally for its 167 million citizens. The COVID-19 Vaccines Global Access (COVAX) allocated 11 million doses of COVID-19 vaccine for Bangladesh and only a few of them have arrived in Bangladesh [21]. While Bangladesh seriously struggles to address its COVID-19 vaccine crises, it is almost impossible for the country to provide vaccinations for Rohingya refugees. Moreover, due to India’s export ban of the COVID-19 vaccine, COVAX may not supply allocated doses of vaccines immediately to Bangladesh, thereby implying that some of the world’s most devastated refugees are at a heightened risk.

Under these realities, international stakeholders, particularly vaccine-producing countries such as the USA, China, UK, and Russia should step in to ensure that the Rohingya refugees who are most susceptible to the COVID-19 are given priority in vaccinations. There are some reasons to be hopeful for Rohingya refugees as most of these countries have immunized a significant number of their citizens. Any single country or all of these stakeholders are capable of immediately ensuring adequate COVID-19 vaccines for the refugees. A recent donor summit of G7 countries secured pledges for 1 billion doses of COVID-19 vaccines for poor countries [22]. Furthermore, the USA alone is ready to donate millions of doses of COVID-19 vaccines through bilateral treaties. Indubitably, these are steps in the right direction to fight against COVID-19, but there is a lack of commitment to ensure adequate doses of COVID-19 vaccine for 26 million refugees worldwide including the most vulnerable 1.3 million Rohingya refugees. Therefore, it is the moral responsibility of vaccine-producing and/or G7 countries to take urgent initiatives to provide adequate doses of COVID-19 vaccines for refugees and to ensure the immediate rollout of vaccination in the Rohingya refugee camps.

## SPECIFIC RECOMMENDATIONS

There should some specific recommendations to handle the Rohingya population before the conditions of these refugee camps touch the point of no return.

1. The COVID-19 preparedness of Rohingya refugees should be considered as special circumstances of a population devastated by prior genocide, leaving homes and wealth, with psychological torture.
2. There should be special COVID-19 cell only for health education, case identification, and management of this population. The barrier of language, education status, and realizing health need should be considered for proper planning.
3. They should get anti-SARS-CoV-2 vaccine as preferential basis because if the vaccine delivered based on the principle of distribution in Bangladesh, these vulnerable group would be endangered. All Rohingya population above the age of 12 years should be immediately vaccinated.
4. As the movement of these people are restricted, the assessment of SARS-CoV-2 and management strategies should be provided by creating field hospitals adjacent to their camps. These facilities should have abundant oxygen supply and requisite drugs. Also, serious and complicated patients should be moved to tertiary treatment center at the district head quarter by special transportation. Adequate numbers of health personnel including physicians should be provided to ensure quality services.
5. In case of fatality, proper measures must be ensured for funeral.

## CONCLUSIONS

The COVID-19 pandemic has highlighted clear discriminatory limitations on access to health care facilities particularly hospital beds, oxygen supplies, intensive care capacities and vaccination for Rohingya refugees. Preparedness for COVID-19 in the refugee camps are affected by the lack of human resources, inadequate management, and laboratory and hospital facilities for testing and treating COVID-19. Bangladesh, like many other resource constrained countries hosting millions of stateless people, needs to strengthen the treatment and vaccination facilities for refugees. However, it is almost impossible for Bangladesh to ensure such facilities for the huge number of Rohingya refugees. Therefore, it is essential for the government to work with international stakeholders and experts on refugee health to take urgent steps to ensure adequate medical facilities and vaccines against COVID-19 to protect the most devastated Rohingya people. Failure or delay to put in these efforts would leave these vulnerable populations dangerously unprotected.
